# Influence of Problematic Mobile Phone Use on Social and Assertiveness Skills in Adolescents

**DOI:** 10.3390/bs16010001

**Published:** 2025-12-19

**Authors:** Juan Carlos Dobado-Castañeda, Verónica Marín-Díaz, Begoña Esther Sampedro-Requena

**Affiliations:** Department of Education, Faculty of Education Sciences and Psychology, University of Cordoba, 14071 Córdoba, Spain; ed1madiv@uco.es (V.M.-D.); bsampedro@uco.es (B.E.S.-R.)

**Keywords:** adolescents, mobile phones, problematic technology use, social skills, assertiveness

## Abstract

Smartphones have become the “backbone” of the connected society, reshaping social interactions in a period of adolescence marked by a neuropsychology vulnerability that is sensitive to intensive technological mediation. This study analyzes the relationship between problematic mobile phone use and the social and assertiveness skills of adolescents. Through a cross-sectional design, the answers of 1864 adolescents aged between 11 and 21 years old from education centers located in Cordoba (Spain) were analyzed, through a questionnaire that collected sociodemographic variables, the MPPUSA scale, to measure the inadequate use of mobile phones, and the ADCA-1 to assess social skills and assertiveness. The results revealed inadequate levels of mobile phone use and low levels of social skills, with nomophobia and negative consequences emerging as the dimensions most related to these outcomes. A decision tree analysis identified negative consequences as the variable with the strongest association with the level of social development. The findings point to a concerning situation in which not only the usage time but also quality of use are associated with the psychosocial development of this population group. Therefore, preventive and educational interventions addressing digital literacy, management of emotions, and the promotion of face-to-face social skills are necessary.

## 1. Introduction

In the last few decades, mobile devices have evolved from simple communication tools to become what some authors have identified as the authentic “backbone” of the connected society, a metaphor that emphasizes their central role in mediating—rather than determining—contemporary social interactions (Alencar & da Rocha, 2020; Campbell & Olteanu, 2024; Fortunati, 2023; Garcia-Swartz & Campbell-Kelly, 2022; Rahman & Islam, 2022). This transformation is especially pronounced during adolescence, a critical stage in which mobile technology not only facilitates communication, but also fundamentally redefines the social frameworks of interaction, belonging, and identity (Avci et al., 2025; ITU, 2022; UNESCO, 2023).

The magnitude of this transformation is reflected in statistical data: while in Spain 94% of the population has a mobile phone, 83.8% has a smartphone (INE, 2023), and worldwide, the number of unique mobile phone users ascends to 5.610 billion, 69.4% of the world’s population (Kemp, 2024). However, these figures barely reveal the surface of a much more complex phenomenon that has redefined the paradigm of “being an adolescent” in the 21st century.

Understanding why this population is particularly affected requires examining the developmental characteristics of this stage of human development. Adolescence is an especially sensitive period to the influx of mobile technologies, due to their role in the development of personal autonomy, the construction of the concept of self, and the interaction among equals (Boyd, 2014; Pastor et al., 2022; Vanden Abeele et al., 2018). What these authors name “connected presence” transcends the simple ability to communicate to become a state in which the borders between physical and digital spaces become progressively blurred.

This connected presence has solidified into what Powell (2017) conceptualizes as a “culture of permanent connectivity”, characterized by social expectations of immediate responses that the youth have naturalized as everyday relationship dynamics. This culture is closely linked to Fear of Missing Out (FOMO), a psychological phenomenon that creates a persistent anxiety about missing relevant social interactions or information, promoting compulsive checking of digital content and exacerbating frequent connectivity (Rathod et al., 2022; Wacks & Weinstein, 2021; Weinstein, 2023). The changes in interpersonal communications are one of the most notable aspects of this revolution, where instant messaging applications, social networks, and video calling platforms, have greatly substituted the traditional methods of communication (Avelar & Rodríguez Pittí, 2022; Johnson et al., 2019).

Within this context of digital transformation, a critical question emerges: when does intensive mobile phone use become problematic? Problematic use is defined as a pattern of behavior characterized by a functional or emotional dependence towards the device, which negatively interferes in daily activities, psychological well-being, and social relations (Olivella-Cirici et al., 2023; Sohn et al., 2021; Wacks & Weinstein, 2021).

To operationalize this construct, the scientific literature has identified a set of clinical manifestations associated with this pattern of problematic use. One of the tools used is the scale adapted to the Spanish population, the MPPUSA (Mobile Phone Problem Use Scale for Adolescents) (López-Fernández et al., 2012). Although not originally composed by specific dimensions, it has been consistently structured around four factors in international studies (García-Umaña & Córdoba Pillajo, 2020; Marín Díaz et al., 2018; Mohammadi Kalhori et al., 2015): “nomophobia” (no-mobile-phone phobia), describing the irrational fear of not having access to the mobile device (Avelar & Rodríguez Pittí, 2022; Chang et al., 2025; Navas-Echazarreta et al., 2023); difficulties in “disconnecting”, conceptualized as an inability to voluntarily regulate device usage time (Domoff et al., 2025); perceived negative consequences, which are thought to shape the third dimension, including academic problems, family conflicts, sleep alterations, and deterioration of interpersonal relations (Azzaakiyyah, 2023; Olivella-Cirici et al., 2023); and “social criticism”, reflecting awareness about concerns raised by their pattern of use in their close environment (Fu et al., 2020; Jungselius, 2024). Recent studies have also identified that individual factors such as age, gender, and ability to regulate emotions may influence vulnerability to these problematic patterns (Azzaakiyyah, 2023; Sohn et al., 2021).

Beyond these psychological dimensions, a fundamental aspect to understand the problematic mobile phone use is recognizing that the patterns of use are not homogeneous throughout the week. Current studies have documented significant differences between the usage time during school days (Monday through Friday), and the weekend (Saturday and Sunday), associated with changes in the academic routines, digital leisure, and the type of activities performed with the device (Olivella-Cirici et al., 2023; Smahel et al., 2020).

This temporal variability is especially relevant, as the contexts of use have a decisive impact on the influence of mobile technologies could have on socio-communicational competences. On school days, the use of mobile phones can more directly interfere with academic activities or structured social interactions, while during the weekend, their prolonged use can displace face-to-face leisure activities and quality family time (Fu et al., 2020; Kuyucu, 2021; Powell, 2017).

The consequences of problematic mobile phone use extend to multiple domains of adolescent well-being, including mental health correlates such as depression, anxiety, and sleep alterations (Andersen et al., 2023; Valkenburg, 2022; Wacks & Weinstein, 2021). In the social dimension, paradoxically in opposition to virtual hyperconnectivity, an excessive use of smartphones can lead to a substantial deterioration of face-to-face social interactions. Adolescents with a problematic pattern of use show a marked preference for communication mediated by technology, to the detriment of face-to-face social interactions (Agnihotri, 2023; Johnson et al., 2019). This prioritization of digital communication can create significant deficits in fundamental skills, such as the maintenance of visual contact, the recognition of facial expressions, and the understanding of body language (Flórez-Madroñero & Prado-Chapid, 2021; Genç & Coskun, 2020; Mammadzada Sevinj, 2023).

However, the complexity of this phenomenon is evidenced by the fact that not all adolescents are equally vulnerable to developing these problematic usage patterns. Research suggests that youth with lower levels of emotional regulation or with more fragile socio-emotional competences have a greater propensity toward dysfunctional smartphone use (Azzaakiyyah, 2023; Sohn et al., 2021). This differential vulnerability highlights the bidirectional nature of the relationship between individual characteristics and technology use: pre-existing deficits in emotional and social skills may predispose adolescents to problematic use, which in turn exacerbates difficulties in socio-communicative competences that are crucial during this stage of development.

Socio-communicative skills constitute a complex and multidimensional set of cognitive, emotional, and behavioral abilities that allow individuals to efficiently establish, maintain, and manage interpersonal interactions in diverse social contexts (Flórez-Madroñero & Prado-Chapid, 2021; Grover et al., 2020). During adolescence, these skills acquire a special relevance, as it is precisely at this stage when peer groups acquire a main role in social validation and the development of identity (Livingstone & Stoilova, 2021; Molleman et al., 2022). Within the broad spectrum of social competences, assertiveness occupies a central place due to its power to integrate of interpersonal and intrapersonal aspects. García Pérez and Magaz Lago (2011), through their ADCA-1 (Self-Report of Attitudes and Values in General Social Interactions), have proposed a two-dimensional taxonomy—self-assertiveness and hetero-assertiveness—which allows this competence to be operationalized in a precise and culturally adapted manner.

However, the expression and development of these assertive competences is increasingly influenced by digital contexts. In modern digital society, technology-mediated social interaction poses specific challenges: digital communication frequently lacks essential non-verbal elements (facial expressions, visual contact, proxemics, paralanguage), which increases the probability of misunderstandings and can lead to interpersonal conflicts that are particularly challenging for adolescents with limited social skills (Agnihotri, 2023; Campbell & Olteanu, 2024; Chang et al., 2025; Mammadzada Sevinj, 2023). Johnson et al. (2019) or Livingstone and Stoilova (2021) point out an interesting paradox: on the one hand, digital settings can be “safer” for some shy adolescents or those with social anxiety, as they provide them time to think and re-formulate messages. On the other hand, this same convenience can make difficult the adequate development of assertive skills, especially in the youth who are more exposed to high-pressure digital contexts or constant social validation (Lamash et al., 2024).

Taken together, the literature highlights a complex interplay between problematic mobile phone use and the development of socio-communicative competences during adolescence. Within this framework, assertiveness emerges as a particularly relevant competence, as it plays a key role in healthy socioemotional development at a stage where technology is reshaping the contexts in which interpersonal skills are learned and expressed. Despite previous evidence linking problematic technology use to diminished social skills, further research is needed to clarify how specific dimensions and temporal patterns of problematic use differentially relate to distinct components of assertiveness, and to identify predictive profiles that may guide targeted educational actions.

## 2. Materials and Methods

### 2.1. Study Design

The present study utilized a cross-sectional ex post facto design, characterized by the recollection of data at a specific moment in time and the observation of already-manifested phenomena without experimental manipulation. This design was framed within an empirical-analytical method, based on the systematic collection of data and their subsequent analysis through advanced statistical techniques. The study included a descriptive, comparative, correlational, and classificatory approach. The descriptive approach allowed for detailing information about mobile phone use and socio-communicative competences. The correlational approach enabled examining possible relationships between these variables and the classificatory approach facilitated identifying user profiles based on the variables studied.

### 2.2. Objectives and Hypothesis

The main objective of the present study was to analyze the relationship between problematic mobile phone use and the degree of development of socio-communicative skills in adolescents. For this, the intention is to identify technology use patterns and the dimensions of problematic use that are most strongly associated with the level of social competences in this population, especially regarding assertiveness.

Based on the theoretical foundations described, and to provide an answer to the objective set, the following hypotheses are posed:

**H1.**  *There is a negative and statistically significant relationship between the level of problematic mobile phone use and the degree of socio-communicative skills in adolescents.*

**H2.**  *The specific dimensions of problematic mobile phone use are negatively and differentially correlated with the dimensions of assertiveness.*

**H3.**  *There are statistically significant relationships between the mobile phone usage time and the scores and level of assertiveness, with these being lower for those who show a more intense use.*

**H4.**  *There will be statistically significant differences in some dimensions of assertiveness as a function of the number of hours of mobile phone use.*

**H5.**  *Some specific dimensions of problematic mobile phone use and time or usage patterns will act as discriminant variables of the level of social skills in adolescents.*

### 2.3. Participants, Ethical Approval and Informed Consent

For the purposes of this study, adolescence was conceptualized as a developmental period spanning from 11 to 21 years of age. This age range acknowledges that adolescence encompasses not only biological maturation but also social and cultural dimensions (Malagón-Terron, 2019). While the World Health Organization (WHO, 2011) defines adolescence as 10–19 years of age, other researchers expand this range. Following the widely accepted three-stage classification—early (10–13 years), middle (14–17 years), and late adolescence (18–21 years) (Palfrey & Gasser, 2020)—the present study adopted the 11–21 years range, with the lower boundary coinciding with Compulsory Secondary Education (E.S.O.) in Spain.

Respect to sample selection, a non-probabilistic convenience sampling approach with purposive selection was employed, consistent with standard practice in school-based population studies. Although traditionally considered to limit generalizability, reliability can be comparable to probability sampling when specific conditions are met (Etikan et al., 2016; Stratton, 2021, 2023). Regarding the selection of educational institutions, participation requests were initially sent to multiple schools. From those that agreed to participate, a purposive selection was conducted to ensure diversity across the following contextual criteria: (a) school type (public and publicly funded private institutions), (b) socioeconomic context (high and medium-low), (c) geographical location (urban and peripheral areas), (d) school size, and (e) institutional policies regarding mobile phone use. This selection process resulted in eight participating institutions.

Furthermore, this study incorporated a large sample size, use of validated instruments, explicit inclusion/exclusion criteria, and participation rates. The inclusion criteria were (a) age between 11 and 21 years old; (b) possession and frequent use of a mobile phone; (c) signed informed consent (from parents/legal guardians for minors, or from participants themselves if adults); (d) Spanish language proficiency; and (e) lack of cognitive diagnostics that impede understanding the instruments. The exclusion criteria included the absence of any of the previous requirements or declining to voluntarily participate. While randomization absence limits strict statistical generalization, these safeguards enhance external validity for comparable educational populations.

From an initial pool of 2085 eligible students, 30 (1.4%) did not participate due to lack of parental consent for minors, voluntary refusal by adult students, or absence on the day of data collection, resulting in 2055 adolescents who agreed to participate (participation rate: 98.6%). Subsequently, 166 participants (8.1%) were excluded for not meeting the inclusion/exclusion criteria, yielding a sample of 1889 adolescents. Finally, outlier detection was performed using the interquartile range (IQR) method with box plots, resulting in a final analytical sample of 1864 adolescents. The final sample comprised 52.2% females and 47.8% males, aged between 11 and 21 years (*M* = 15.52; *SD* = 2.32). Participants were enrolled in public (49.8%) and publicly funded private institutions (50.2%), pursuing Compulsory Secondary Education (E.S.O.) (52.3%), Baccalaureate (22.8%), Basic Vocational Training (4.9%), or Medium/Higher Vocational Training (20.0%).

This study was conducted under institutional and departmental supervision at the University of Córdoba. All procedures were performed in accordance with the ethical standards established in the Declaration of Helsinki (World Medical Association, 2013) and the Ethical Principles of Psychologists and Code of Conduct (American Psychological Association [APA], 2017).

Likewise, a written informed consent form was created directed to adult participants and the parents or legal tutors in the case of the minors. The informed consent clearly explained the research objectives, the purpose of data collection, the voluntary nature of participation, the right to withdraw at any time, and the measures implemented to ensure confidentiality and anonymity, in compliance with Organic Law 3/2018 (Agencia Estatal Boletín Oficial del Estado, 2018), of 5 December 2018, on the Protection of Personal Data and the Guarantee of Digital Rights, in addition to Regulation (EU) 2016/679 of the European Parliament and of the Council of 27 April 2016 (The European Parliament and the Council of the European Union, 2016), on the protection of natural persons with regard to the processing of personal data and on the free movement of such data.

### 2.4. Research Instruments and Variables

The questionnaire used for the present study was composed of three differentiated parts or dimensions that as a set constituted a complete instrument. The first part consisted of by sociodemographic variables that collected data on gender, age, type of education center, degree in progress, specialty, mobile phone usage time per week and per weekend, and types of mobile phone use.

The second part evaluated the inadequate use of mobile phones by adolescents. For this, the Spanish adaptation for an adolescent population (MPPUSA) (López-Fernández et al., 2012) of the Mobile Phone Problem Use Scale (MPPUS) (Bianchi & Phillips, 2005) was used. This is a psychometric tool designed to evaluate the levels of problematic or potentially addictive use with respect to the use of mobile phones among Spanish youth. The original MPPUSA was composed of 27 items, with the final version composed of 22 items after the corrected homogeneity analysis of each of them, which possessed a Likert-type response model from complete disagreement (1) to complete agreement (5). After the Exploratory Factor Analysis (EFA) with fit indices KMO = 0.92 and Bartlett’s sphericity test with *p* < 0.001, four factors were found: “nomophobia” (7 items; α = 0.753), referring to the anxiety experienced when lacking mobile phone access, “disconnection” (5 items; α = 0.751), reflecting the inability to reduce mobile phone use, “negative consequences” (7 items; α = 0.703), addressing adverse economic, psychosocial, and physical effects, and “social criticism” (3 items; α = 0.761), concerning perceived excessive use by oneself and one’s social environment. As for the reliability, it was measured through the use of the internal consistency approach, with a Cronbach’s alpha value of 0.884 obtained for the entire set, which demonstrates the high reliability of the instrument.

Neither the original MPPUS nor its Spanish adaptation (MPPUSA) for adolescents established clinically validated cutoff points for score classification. Furthermore, technological and behavioral patterns have changed substantially over the past decade, with problematic smartphone use prevalence increasing from approximately 23% to over 35% globally (Sohn et al., 2019; Lu et al., 2024), which questions the applicability of outdated norms. Given these considerations, the present study adopted a quartile-based approach (P25, P50, P75), a methodologically sound classification recommended in psychometric research and employed in numerous studies when standardized norms are unavailable or of limited applicability to the target population (Cohen & Swerdlik, 2018; Crawford & Garthwaite, 2009; Schmidt & Dirkin, 2022). The resulting classification was: “Occasional” (22–43 points), “Habitual” (44–54 points), “At risk” (55–65 points), and “Problematic” (>65 points). While this sample-dependent categorization may limit direct comparability with other populations, it provides a robust framework for examining prevalence and associated factors based on the actual distribution of the sample.

In the third part of the instrument, the ADCA-1 questionnaire was used (García Pérez & Magaz Lago, 2011). This questionnaire evaluates attitudes, values, and behaviors related with social competence and assertiveness of adolescents. The ADCA-1 is composed of 35 items, of which the first 20 belong to the subscale “Self-assertiveness”, defined as the degree to which an individual respects and acts in accordance with their own rights, thoughts, feelings, tastes, and desires during social interactions, and the following 15 to the subscale “Hetero-assertiveness”, defined as the degree to which an individual respects and considers the rights, thoughts, feelings, tastes, and desires of others in interpersonal situations. The response options utilize a Likert scale from never to almost never (1) to always or almost always (4). Raw scores on each subscale were converted to percentile scores using the age and educational level-specific norms provided in the test manual. Since the manual does not provide normative data for the total score, overall social skills level was classified using a quartile-based approach (P25, P50, P75) derived from the study sample.

### 2.5. Procedure and Data Collection

Firstly, a comprehensive questionnaire that included sociodemographic variables and the MPPUSA and ADCA-1 variables was created. In addition, the corporate logos of the university were added, as well as an explanatory text about the objectives, the instructions on how to complete it, and the estimated time needed to complete it.

Several educational institutions were contacted through a formal letter explaining the aims and objectives of the study, the research team composition, ethical considerations, anonymity guarantees, the voluntary nature of participation, and data management and storage procedures. Once institutional authorization was obtained, eight institutions were selected, and informed consent was sent to these institutions for distribution to adult participants and to families in cases where students were minors. Once all signed consent forms were returned, the research team proceeded to enroll or exclude the participating adolescents.

Lastly, the research team coordinated with the management and/or guidance department of each center to schedule in-person data collection during regular school hours. To ensure consistency and minimize potential biases, all questionnaires were administered by the same researcher across all participating institutions. A standardized protocol was implemented, consisting of: (a) a brief introduction of the researcher and explanation of the study’s purpose; (b) detailed instructions on how to complete the questionnaire, emphasizing the importance of honest responses; (c) clarification of any doubts raised by participants; and (d) distribution of printed questionnaires. To reduce potential social desirability bias in adolescent self-reports, the researcher left the classroom after providing instructions, while a designated teacher remained present to supervise the session and ensure proper completion of the questionnaires. This procedure was designed to balance standardization with participant comfort and anonymity. Participation was completely voluntary, anonymous, and without compensation.

### 2.6. Data Analysis

The data were analyzed using the IBM SPSS Statistics^®^ (version 24) and Jamovi^®^ (version 2.4.14) software programs. A level of significance of *p* < 0.05 was established in all the statistical tests. As a preliminary analysis, the normality of the distributions was examined through asymmetry coefficients and kurtosis, and the atypical and extreme cases of the main variables of the study were identified and eliminated.

Afterwards, a descriptive analysis was performed to calculate the measurements of central tendency, dispersion, and distribution of the frequencies of the variables. Then, through inferential analyses, a one-way analysis of variance and correlations were used to discover differences and identify associations. Lastly, to predict and classify homogeneous groups in the sample, a decision tree analysis was performed using the CHAID (Chi-squared Automatic Interaction Detection) algorithm.

## 3. Results

### 3.1. Time and Types of Smartphone Use by Adolescents

The descriptive analysis of the time spent with the mobile phone revealed differentiated patterns between the weekdays (considered as Monday to Friday) and the weekend (Saturdays and Sundays). The mean obtained during the week was 2.26 h per day (*SD* = 1.18), where 61.1% of the participants reported a usage time between 0 and 25 h, and 38.9% at least 26 h. In addition, during the weekend, the mean usage time increased to 2.39 h per day (*SD* = 1.07). Of the participating adolescents, 49.4% affirmed using their phones for 13 or more hours on Saturdays and Sundays (almost half of the participating sample). Table 1 shows the frequencies and percentages distributed according to categories of usage time for both periods of time.

**Figure 1:**
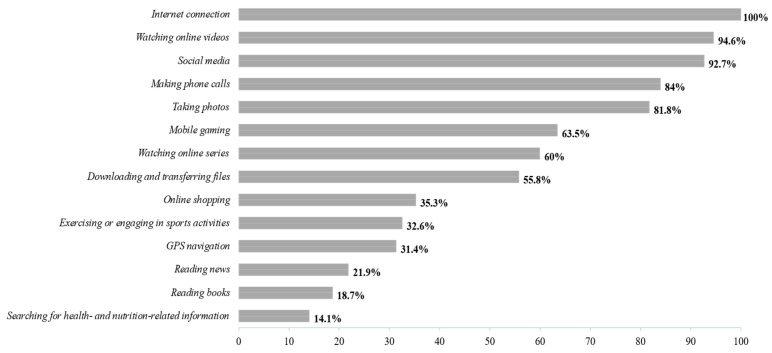
Percentages of types of mobile phone use according to adolescents’ preferences. Note: Prepared by the authors.

As for the activities or types of specific uses of the mobile phone (Figure 1), the data indicated that the adolescents mainly preferred the use and consumption of applications of social character and instant messaging, just as the consumption of visual multimedia content. More than 90% used their mobile device to communicate or entertain themselves, while other uses were within the area of academics, reading, or healthy habits, showed lower frequencies.

### 3.2. Normality, Distribution and Descriptions of the Main Study Dimensions

The Kolmogorov–Smirnov test used to analyze the normality of the main dimensions of research did not meet this assumption (*p* < 0.05). this result is expected given the large sample size (*N* = 1864), as this test is overly sensitive and frequently rejects normality even when distributions are acceptable for parametric analyses (Bilon, 2023). Therefore, complementary distributional diagnostics were examined. Skewness values ranged between −0.5 and 0.5 for all dimensions except “Negative consequences”, which showed a slight positive skew indicating higher scores. Kurtosis values fell within the −1 to 1 range. Additionally, visual inspection of Q–Q plots revealed that observed quantiles closely followed the theoretical diagonal line, with only minor deviations at the extremes. These results suggest approximate normal distribution across the study variables (Pardo Merino, 2010). Likewise, the histograms displayed unimodal and roughly symmetric shapes consistent with a normal distribution. Taken together, this multi-criteria evaluation supports that the normality assumption can be reasonably considered fulfilled despite the significant Kolmogorov–Smirnov statistics.

In order to present a general overview of the data, the descriptive statistics corresponding to the dimensions of the scales used in the study were included: MPPUSA and ADCA-1 (Table 2). The minimum, maximum, means and standard deviation observed in each dimension are indicated, which allows characterizing the distribution of responses in the total sample. This information is pertinent for contextualizing the posterior analyses and to assess the dispersion and central tendency of the data utilized in the inferential tests.

**Figure 2:**
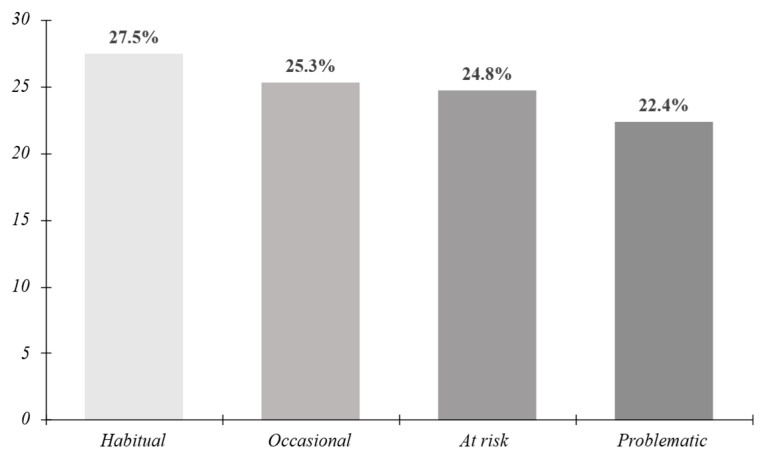
Percentage representation of mobile usage levels according to the scores obtained. Note: Prepared by the authors.

For the MPPUSA scale, after the exploratory factor analysis, a structure with four factors was confirmed, with adequate fit indices (KMO = 0.921; χ^2^ (231) = 10,935.6, *p* < 0.001). The final scale, composed of 22 items after the elimination of five elements with a low homogeneity, showed a satisfactory internal consistency (α = 0.884). With respect to the scores obtained in the questionnaire, a classification was created according to the level of mobile phone usage. Figure 2 shows that the percentages were very similar in all the groups, placing 52.8% of the participants within an acceptable use, and on the contrary, 47.2% within an inadequate or problematic use of the mobile phone.

**Figure 3:**
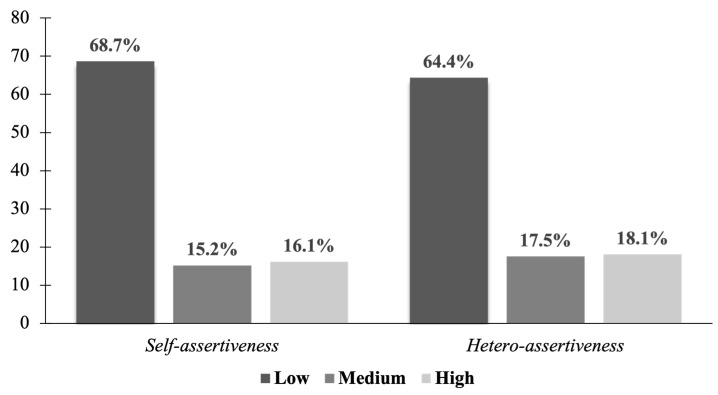
Percentage distribution of self-assertiveness and hetero-assertiveness levels among participants. Note: Prepared by the authors.

Regarding the ADCA-1 questionnaire, participants were classified into three levels (low, medium and high) for each subscale, as presented in Figure 3. For both self-assertiveness and hetero-assertiveness, more than half of the participants were classified at the low level. With respect to the overall level of social skills, half of the sample (50.6%) exhibited low social skills, while 25.9% showed medium levels and 23.6% demonstrated high social skills.

### 3.3. Differences in Assertiveness According to the Mobile Phone Usage Time

The analysis of variance (ANOVA) performed to compare the dimensions of the ADCA-1 scale as a function of mobile phone usage hours revealed statistically significant differences. Levene’s test confirmed the homogeneity of variances for all ADCA-1 dimensions in both time periods (*p* > 0.05), supporting the appropriateness of parametric ANOVA tests.

During the week, Table 3 shows that “Hetero-assertiveness” showed differences between the usage groups (*F* (3, 1860) = 3.272, *p* = 0.020, *η*^2^ = 0.005), with a lower mean score observed among the adolescents who used the mobile phone for more than 35 h (*M* = 1.47; *SD* = 0.52), as compared to those who used it between 0 and 15 h (*M* = 1.54; *SD* = 0.55). No significant differences were found in “Self-assertiveness”, and with respect to the total score, a decreasing trend was observed as the number of usage hours in-creased. Although the differences reached statistical significance due to the large sample size, the effect sizes were small. These findings should be interpreted with caution from a practical standpoint.

During the week, “Self-assertiveness” did not show statistically significant differences (*p* = 0.174). On the contrary, “Hetero-assertiveness” once again showed significant differences (*F* (3, 1860) = 3.857, *p* = 0.009, *η*^2^ = 0.006), as well as the total score obtained (*F* (3, 1860) = 3.265, *p* = 0.021, *η*^2^ = 0.005), in which just as in the previous case, a progressive decrease in the score was observed as the usage time increased (Table 4). The adolescents who used the mobile phone less during the weekend (0–8 h) showed better levels of hetero-assertiveness and social skills in general, as compared with those who used it for a longer period of time (13–20 h and >20 h). Similarly to the previous analyses, the usage patterns over the weekend showed small effect sizes.

### 3.4. Relationship Between the Dimensions of Problematic Mobile Phone Use and Attitudes and Values in Social Interactions

The Pearson’s correlation analysis showed inverse and statistically significant relationships between problematic mobile phone use and social skills (Table 5). Moderate negative correlations were observed between the total score of problematic mobile phone use and the dimensions of assertiveness (*r* = −0.338 with the total score of social skills, *p* < 0.001). More specifically, the dimension “Nomophobia” showed small-to-moderate correlations with “Self-assertiveness” (*r* = −0.296) and “Hetero-assertiveness” (*r* = −0.297), followed by “Negative consequences” (*r* = −0.266 and *r* = −0.232, respectively). These findings suggest that a higher level of dependency or dysfunctional use of the mobile phone is associated with a weaker development of the socio-communicative competences.

### 3.5. Analysis of Classificatory Variables for the Level of Social Skills Using Decision Trees

To identify the key variables that best discriminate social skill profiles, a CHAID decision tree analysis was performed. The algorithm was configured with the following parameters: (a) minimum size of the parent node = 100 cases, (b) minimum size of the child node = 50 cases, (c) automatic maximum depth, (d) level of significance α = 0.05 with the Bonferroni correction, and (e) Pearson’s chi square criterion for the selection of divisions. The CHAID algorithm uses significance-based stopping rules, thus no additional pruning was required. A ten-fold cross validation was applied to assess the stability of the model.

**Figure 4:**
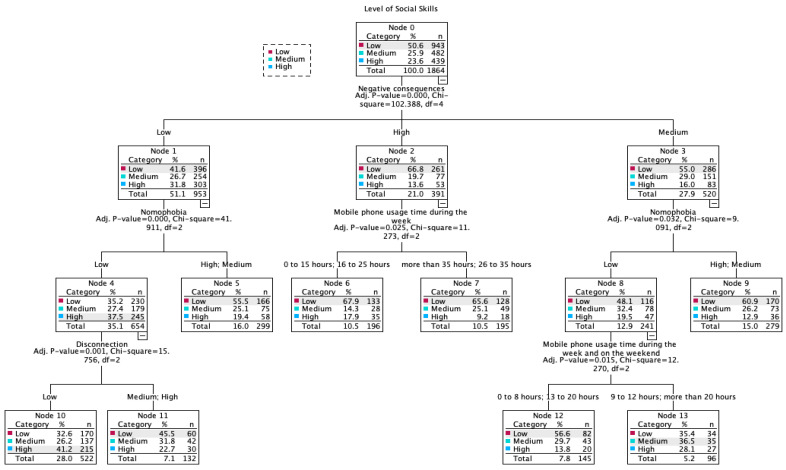
Decision tree of the degree of social skills according to classificatory variables.

The dependent variable was the degree of social skills, categorized into three ordinal levels (low, medium, and high). The independent variables included the sociodemographic indicators (gender, age category, type of center, degree, grouped specialty) and the four psychometric dimensions of problematic mobile phone use. The final model had an architecture composed of 14 nodes (8 terminal nodes) with a maximum depth of 3 levels (Figure 4). Model validation yielded a risk estimate of 0.348 (SE = 0.012), indicating an overall classification accuracy of 65.2%. Of the eleven variables introduced, the algorithm identified five significant discriminant variables: “Negative consequences” (primary splitting variable), “Nomophobia”, time of phone usage during the week, “Disconnection”, and hours of phone use on the weekend.

The dimension “Negative consequences” emerged as the primary splitting variable (χ^2^ = 102.388, *p* < 0.001), establishing the main partition of the tree and identifying key patterns in the levels of social skills. This variable generated three main branches that distributed the sample into: low (50.6%, *n* = 943), medium (25.9%, *n* = 482), and high (23.6%, *n* = 439) consequences. The secondary subdivisions were structured hierarchically:

- In the branch of low negative consequences: nomophobia constituted the second level of division, differentiating between individuals with low vs. high/medium nomophobia.
- In the branch of high negative consequences: the time spent using the phone during the week segmented the users between patterns of moderate use vs. intensive use.
- In the branch of medium negative consequences: once again nomophobia acted as a secondary discriminant variable.

The analysis revealed consistent differential patterns on the problematic use of technology in two profiles:

- A lower social skills profile that included individuals with high negative consequences associated with the use of the mobile phone, high levels of nomophobia, and patterns of intense use, who tended to predominantly be classified as having a low or medium degree of social skills.

A profile with high social skills that was characterized by individuals with low negative consequences derived from the use of the mobile phone, reduced levels of nomophobia, a decreased usage time during the week, low disconnection, and a low number of hours of phone use during the weekend.

## 4. Discussion

The findings provide support for the study hypotheses. Specifically, H1 and H2 were accepted. H3 received partial support, as statistically significant differences in hetero-assertiveness were found according to usage time, although effect sizes were small. Similarly, H4 was partially accepted, with significant differences observed only in hetero-assertiveness, but not in self-assertiveness. Finally, H5 was accepted.

In line with these results, the findings obtained in the present study show, through statistically significant associations of small to moderate magnitude, the existence of a negative and consistent relationship between problematic mobile phone use and the development of social skills in adolescents, and therefore, the objectives and hypothesis posed can be answered (Wan et al., 2022; Yoon et al., 2025). Although effect sizes were modest, these findings have important theoretical and practical implications for understanding these psychosocial changes in a digital era.

Furthermore, the analysis revealed a modest, inverse, and statistically significant relationship between problematic mobile phone use and the socio-communicative skills of the adolescents. Negative correlations of moderate magnitude appeared in all the dimensions of mobile phone use and social skills of assertiveness. This finding is consistent with theories such as the social displacement theory (Przybylski & Weinstein, 2017) or competency atrophy (Uhls et al., 2014), due to the deterioration of face-to-face social interactions, the development of social anxieties, or external validation dependence (Olivella-Cirici et al., 2023; Wacks & Weinstein, 2021).

From a neuropsychological perspective, the interpretation is that the intensive interaction with mobile devices during adolescence (a period characterized by the significant reorganization of brain circuits linked with socioemotional processing) can interfere with basal maturation processes for the development of social competences (Casey et al., 2019; Maza et al., 2023).

It is particularly alarming that 47.2% of the participants were found in usage levels classified as “at risk” or “problematic”, significantly exceeding the previous estimations by Sohn et al. (2019) of 23.3% in global meta-analyses, or those by Vaghasiya et al. (2023) with a value of 24.4% in a similar population. This prevalence practically doubles the expectations based on the literature, suggesting a possible intensification of the phenomenon in modern populations, or the existence of specific contextual factors that aggravate the problem.

Complementarily, the finding that more than 50% of the adolescents showed low levels of social skills is an important finding for public health. This proportion indicates that deficient socio-communicative competence has reached levels that exceed individual cases to become a widespread population phenomenon, with potentially lasting implications for psychosocial functioning in the transition to adulthood.

One of the notable findings is that “nomophobia” was presented as a relevant risk factor. This dimension, understood as an irrational fear of not having access to the mobile phone, showed the strongest correlations among the dimensions studied, although these associations remain moderate in absolute terms. Nomophobia may function as an indicator of an underlying social anxiety, limiting the predisposition in participating in face-to-face interactions and reducing the exposure to natural contexts of communicative learning (Chang et al., 2025; Rahman & Islam, 2022). These results can be interpreted in light of the concept of “connected presence” described by Vanden Abeele et al. (2018), where the boundaries between physical and digital spaces progressively blur. The nomophobia identified as a notable risk factor could reflect this internalization of the permanent connectivity described by Powell (2017), where device absence generates anxiety due to the inability to maintain such presence. Furthermore, FOMO could underlie both nomophobia and negative consequences, intensifying compulsive usage patterns (Rathod et al., 2022; Weinstein, 2023).

This pattern suggests a psychological dependence that goes beyond the mere instrumental use, potentially serving as a technological mechanism that interferes in the social development of adolescents. All of this acquires a special relevance considering that the literature has documented that approximately 25% of Spanish adolescents experience nomophobia (Diaz Beloso & Quintanilla, 2024), although the data from the present study suggest more generalized effects on social competences, even in subclinical levels of this dependence.

On the other hand, mobile phone usage time during the week showed a statistically significant, though small-sized, differences with the “hetero-assertiveness” dimension (Klimenko et al., 2024). The adolescents who presented a lower use showed somewhat higher levels in this dimension, as compared to those who had a more intense use. This relationship is consistent with the exposure time as another critical factor, although the modest effect size indicates that usage time explains a proportion of the variance in social skills. Neurological models of behavioral sensitization suggest that a prolonged exposure to gratifying digital stimuli can generate neuroplastic adaptations that increase de-pendency and reduce the sensitivity to non-digital reinforcers.

An interesting finding is that hetero-assertiveness showed greater sensitivity to mobile phone usage time than self-assertiveness. This differential pattern could be explained by the nature of digital interactions: while defending one’s own rights (self-assertiveness) can be practiced in both digital and face-to-face contexts, the ability to respect and consider others’ rights (hetero-assertiveness) may require more complex social cues that are often absent in technology-mediated communication (Agnihotri, 2023; Campbell & Olteanu, 2024). In this regard, the paradox described by Johnson et al. (2019), whereby digital environments may feel safer for socially anxious adolescents but simultaneously hinder assertive skill development, aligns with these findings. Digital contexts may facilitate expressing one’s own positions while limiting opportunities for practicing the recognition of others’ perspectives that rely on non-verbal cues (Livingstone & Stoilova, 2021; Mammadzada Sevinj, 2023).

Due to this, it is worrying that 38.9% of the participants used the mobile phone for 26 or more hours during the week, while 49.4% used it for 13 or more hours during the weekend. These data indicate that approximately half of the sample exceeded the thresholds identified by the scientific literature as critical for healthy development (Wacks & Weinstein, 2021; Zhang et al., 2024).

As for the type of use and preferences of the adolescents, the socio-creative activities predominated. Most of the participants used these devices for social networks and for the consumption of audiovisual contents, as opposed to education-information activities. This pattern suggests the consolidation of a paradigm of use oriented to virtual socialization and entertainment, in detriment to actions that promote the development of critical competences or self-development ones. According to the theory of gratification, it is evidenced that adolescents seek to satisfy their needs of social belonging, entertainment, and identity validation through their smartphones.

The analysis through the use of decision trees identified that the “negative consequences” dimension was the strongest discriminant variable of the level of social skills among the variables examined. This implies that when the use of the smartphone starts to systematically interfere with fundamental day-to-day activities, social development may be negatively affected. It is noteworthy that 66.8% of the participants with high negative consequences simultaneously presented low levels of social skills, while 31.8% of the youth with low negative consequences had high levels of social competences. This differentiation provides preliminary empirical support for the paradigm of “quality over quantity” with respect to the use of technology, suggesting that the qualitative pattern of use may be more determining than the absolute temporal exposure.

Finally, regarding the limitations of this study, the cross-sectional design precludes causal inferences; longitudinal research is needed to clarify the directionality of these relationships. Additionally, the reliance on self-report measures may introduce social desirability biases; future studies could include objective usage metrics and multi-informant approaches. Furthermore, despite the large sample size and methodological safeguards to enhance external validity, convenience sampling and recruitment from a single Spanish province (Córdoba) limit strict generalizability. Nevertheless, the findings may be applicable to comparable educational populations, although caution is advised when extrapolating to contexts with different cultural patterns of digital device use. Moreover, the age range (11–21 years) encompasses heterogeneous developmental stages that may relate differently to technology; future research should examine these associations separately across early, middle, and late adolescence. In final terms, the interpretation of instrument scores relied on sample-derived cut-off points, which may affect comparability with other studies. Despite these limitations, the findings provide valuable insights into the relationship between problematic mobile phone use and social skills in adolescents.

## 5. Conclusions

The present study offers a meaningful contribution to the analysis of the impact of inadequate use of mobile phones on socio-communicative development during adolescence. This influence does not answer to a single factor, but is shaped through a complex and interwoven paradigm of personal, contextual, and technological variables.

First, it is observed that adolescents with a more compulsive and emotional use conditioned by the mobile phone (especially those with high levels of nomophobia and social criticism) manifest statistically significantly, though modestly, lower levels of self-assertiveness and hetero-assertiveness. These results reinforce the need to include a comprehensive perspective that considers psychological aspects related with technology and not only centered on the frequency of use. Second, the adolescent digital ecosystem is characterized by its predominant socio-recreational use, with practically all of them using their mobile devices for social networks and audiovisual content, while the educational uses present a lower prevalence.

Next, more than half of the adolescents presented low levels of social skills, shaping a population phenomenon that transcends individual cases to become a notable adolescent public health concern. In addition, there are identifiable temporal thresholds, with 38.9% of the participants who exceeded 26 weekly hours of use on school days, while 49.4% exceeded 13 h during the weekend, with detectable yet small and consistent dose–response effects in hetero-assertiveness.

Conversely, the predominance of the style of use over exposure was supported by the data, as a predictor of socio-communicative development. The adolescents with a pattern of use that was more reflective, socially constructive, and emotionally self-regulated, tended to have somewhat higher indicators of social competence, even when their usage time was higher. In turn, those who presented a more compulsive use associated with external validation showed greater relational deficits.

## Figures and Tables

**Table 1:** Descriptive data on mobile phone usage time during the week and on weekends.

Variables (*N* = 1864)	*M* (hours/day)	*SD*	Categories	*f*	*%*
Mobile phone usage time during the week (M-F)	2.26	1.18	0 to 15 h	675	36.2
			16 to 25 h	464	24.9
			26 to 35 h	286	15.3
			More than 35 h	439	23.6
Mobile phone usage time during the weekend (Sa-Su)	2.39	1.07	0 to 8 h	524	28.1
			9 to 12 h	419	22.5
			13 to 20 h	597	32
			More than 20 h	324	17.4

Note: *N* = sample size; *M* = mean; *SD* = standard deviation; *f* = frequency; % = percentages; M-F = Monday to Friday; Sa-Su = Saturday and Sunday. Prepared by the authors.

**Table 2:** Descriptive statistics for the dimensions of the scales used in the study.

Scales (*N* = 1864)	Dimensions	Minimum	Maximum	*M*	*SD*
MPPUSA	Nomophobia	1	5	2.77	0.87
	Disconnection	1	5	2.92	0.94
	Negative consequences	1	4.57	1.77	0.63
	Social criticism	1	5	2.72	1.09
	Total score	1	4.82	2.55	0.71
ADCA-1	Self-assertiveness	0.28	3	1.83	0.49
	Hetero-Assertiveness	0	3	1.54	0.54
	Total score	4.50	52.50	29.70	8.08

Note: *N* = sample size; *M* = mean; *SD* = standard deviation. Prepared by the authors.

**Table 3:** ANOVA of the ADCA-1 dimensions in relation to the hours of mobile phone use during the week.

Dimensions	Hours of Mobile Phone Use (M-F) (*N* = 1864)				ANOVA Statistical Values			
	0 to 15 h (*n* = 675)	16 to 25 h (*n* = 464)	26 to 35 h (*n* = 286)	+ than 35 h (*n* = 439)				
ADCA-1	*M/SD*	*M/SD*	*M/SD*	*M/SD*	*F*	*df1*;*df2*	*p*	*η* ^2^
Self-assertiveness	1.85 ± 0.50	1.86 ± 0.49	1.79 ± 0.48	1.80 ± 0.49	1.842	3;1860	0.137	0.003
Hetero-Assertiveness	1.58 ± 0.55	1.54 ± 0.55	1.55 ± 0.52	1.47 ± 0.52	3.272	3;1860	0.020	0.005
Total score	30.15 ± 8.18	29.97 ± 8.19	29.37 ± 7.70	28.92 ± 8	2.411	3;1860	0.065	0.004

Note: M-F = Monday to Friday; *N*/*n* = sample size; *M* = mean; *SD* = standard deviation; *F* = ANOVA statistic; *df* = degrees of freedom; *p* = significance level; *η*^2^ = eta squared. Prepared by the authors.

**Table 4:** ANOVA of the ADCA-1 dimensions in relation to the hours of mobile phone use on weekends.

Dimensions	Hours of Mobile Phone Use (Sa-Su) (*N* = 1864)				ANOVA Statistical Values			
	0 to 8 h (*n* = 524)	9 to 12 h (*n* = 419)	13 to 20 h (*n* = 597)	+ than 20 h (*n* = 324)				
ADCA-1	*M/SD*	*M/SD*	*M/SD*	*M/SD*	*F*	*df1;df2*	*p*	*η* ^2^
Self-assertiveness	1.86 ± 0.49	1.84 ± 0.49	1.81 ± 0.49	1.81 ± 0.50	1.659	3;1860	0.174	0.003
Hetero-Assertiveness	1.59 ± 0.56	1.57 ± 0.52	1.50 ± 0.54	1.49 ± 0.52	3.857	3;1860	0.009	0.006
Total score	30.41 ± 8.19	30.07 ± 7.89	29.12 ± 8.11	29.11 ± 7.97	3.265	3;1860	0.021	0.005

Note: Sa-Su = Saturday and Sunday; *N*/*n* = sample size; *M* = mean; *SD* = standard deviation; *F* = ANOVA statistic; *df* = degrees of freedom; *p* = significance level; *η*^2^ = eta squared. Prepared by the authors.

**Table 5:** Pearson correlation coefficient between the MPPUSA dimensions and the ADCA-1 dimensions.

Dimensions	Self-Assertiveness		Hetero-Assertiveness		Total ADCA-1 Score	
	*r*	*p*	*r*	*p*	*r*	*p*
Nomophobia	−0.296 **	0.000	−0.297 **	0.000	−0.331 **	0.000
Disconnection	−0.208 **	0.000	−0.198 **	0.000	−0.226 **	0.000
Negative consequences	−0.266 **	0.000	−0.232 **	0.000	−0.281 **	0.000
Social criticism	−0.236 **	0.000	−0.223 **	0.000	−0.257 **	0.000
Total MPPUSA score	−0.310 **	0.000	−0.294 **	0.000	−0.338 **	0.000

Note: *r* = Pearson coefficient; *p* = significance level; ** *p* < 0.001. Prepared by the authors.

## Data Availability

The data presented in this study are available upon request to the corresponding author. The data are not publicly available due to ethical and privacy considerations related to safeguarding the confidentiality of minor participants, in accordance with the American Psychological Association’s Ethical Principles of Psychologists and Code of Conduct (Standard 8.14) and as provided by Spanish data-protection legislation, the Organic Law on the Protection of Personal Data and Guarantee of Digital Rights (LOPDGDD).
